# Anointing with Oil

**Published:** 1854-04

**Authors:** 


Anointing with oil.—Professor Simpson, of Edinburg, has been the means of bringing to light a curious corroboration of the sanitary value of the ancient practice of anointing with oil. It appears that the learned professor, when recently visiting the manufacturing town of Galashiels, was casually informed that the workers in the wool-mill in that place were exempt from the attacks of consumption and scrofula. On inquiring of the medical men in the vicinity, the truth of the statement was confirmed, and it was then deemed expedient to pursue investigations on a broader scale. Communications were accordingly sent to physicians residing in Dunfermline, Alloa, Tillicoultry, Inverness, and other districts where the wool-mills are in operation; and in the case of all it was ascertained that similar immunity was enjoyed from the fatal diseases mentioned. It further appeared that, in some of the localities, scarlatina had been added to the list; and, also, that employment in the mills not only preserved health, but children of delicate constitutions were sent to be wool workers, for the express purpose of acquiring strength—a result in almost every instance attained.

